# Author Correction: A simple model for Behavioral Time Scale Synaptic Plasticity (BTSP) provides content addressable memory with binary synapses and one-shot learning

**DOI:** 10.1038/s41467-025-56459-9

**Published:** 2025-01-29

**Authors:** Yujie Wu, Wolfgang Maass

**Affiliations:** 1 Department of Computing, The Hong Kong Polytechnic University, Hong Kong, SAR China; 2 Institute of Theoretical Computer Science, Graz University of Technology, Graz, Austria

**Keywords:** Computational science, Computational neuroscience

Correction to: *Nature Communications* 10.1038/s41467-024-55563-6, published online 2 January 2025

In this article the following sentence was omitted from the acknowledgements section, ‘For open access purposes, the author has applied a CC BY public copyright license to any author accepted manuscript version arising from this submission’. The original article has been corrected.

